# Living donor liver transplantation from a donor previously treated with interferon for hepatitis C virus: a case report

**DOI:** 10.1186/1752-1947-5-276

**Published:** 2011-07-03

**Authors:** Masaaki Hidaka, Mitsuhisa Takatsuki, Akihiko Soyama, Hisamitsu Miyaaki, Tatsuki Ichikawa, Kazuhiko Nakao, Takashi Kanematsu, Susumu Eguchi

**Affiliations:** 1 Department of Surgery, Nagasaki University Graduate School of Biomedical Sciences, 1-7-1 Sakamoto, Nagasaki 852-8501, Japan; 2 Department of Gastroenterology and Hepatology, Nagasaki University Graduate School of Biomedical Sciences, Nagasaki, Japan

## Abstract

**Introduction:**

Selecting a marginal donor in liver transplantation (LT) remains controversial but is necessary because of the small number of available donors.

**Case presentation:**

A 46-year-old Japanese woman was a candidate to donate her liver to her brother, who had decompensated liver cirrhosis of unknown origin. Eight years before the donation, she had a mild liver dysfunction that was diagnosed as a hepatitis C virus (HCV) infection (serotype 2). She had received anti-viral therapy with interferon α-2b three times weekly for 24 weeks and had a sustained viral response (SVR). A biopsy of her liver before the donation showed normal findings without any active hepatitis, and her serum was negative for HCV-RNA. Only 67 patients have undergone LT from a cadaveric donor in Japan. The family in this case decided to have living donor LT. A careful selection for the liver graft donation was made; however, since she was the only candidate, we approved her as a living donor. She was discharged nine days after the liver donation. Her liver function recovered immediately. A computed tomography scan showed sufficient liver regeneration one year later. Her brother also had good liver function after LT and had no HCV infection 48 months after surgery and no *de novo *malignancy. Neither of the siblings has developed an HCV infection.

**Conclusions:**

A patient with SVR status after interferon therapy might be considered a candidate for living donor LT but only if there are no other possibilities of LT for the recipient. A careful follow-up of the donor after donation is needed. The recipient also must have a very close follow-up because it is difficult to predict what might happen to the graft with post-transplant immunosuppression.

## Introduction

The number of deceased donor liver transplantations (DDLTs) in Japan is extremely small. There were 67 cases between February 1999 and January 2010, according to the Japan Organ Transplant Network [1]. Therefore, living donor liver transplantation (LDLT) is the most frequent treatment option for patients with end-stage liver disease in Japan. The main advantage of LDLT over DDLT is that the donor can be completely evaluated, before the operation, to exclude many medical problems. However, the indications for a living donor should be strict and the risk to the donor must be avoided with the greatest care. Donors with possibly morbid liver conditions, including fatty infiltration or a history of viral hepatitis, and older donors offer "marginal grafts", which should be used only after very careful evaluation. A hepatitis B virus (HBV) core antibody seropositive donor can be accepted as long as HBV surface antigen is seronegative and anti-viral treatment is administered to the recipient after transplantation [2,3]. In this way, donor safety also is established, according to several reports of this type of case [4-6]. Some investigators reported that the patients obtained a sustained viral response (SVR) after interferon therapy showed that there was no tendency to develop fibrotic liver in the future [7,8]. HCV-RNA was not detected in 88% of the serum and liver biopsies of patients with an SVR [9]. The infection rate of the recipient from an HCV-positive graft should be low after LT. The rate of carcinogenesis has increased at an annual rate of 0.11% after an SVR was maintained with anti-viral therapy [10]. Using a graft with a positive hepatitis C virus (HCV) antibody although HCV-RNA was not detected in the blood remains controversial in Western countries [11-13]. To the best of our knowledge, there is no actual data that shows the outcome of liver transplantation with a graft from patients who acquire an SVR after successful anti-viral therapy. Here, we report the case of a living donor who had an SVR before LDLT. The case is described and discussed in detail.

## Case presentation

**Figure 1:**
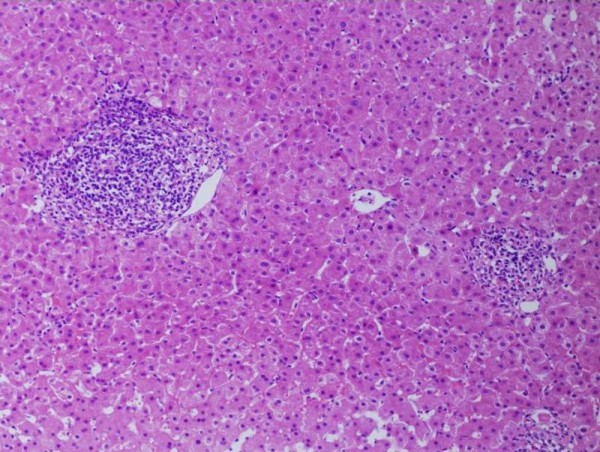
**A liver biopsy showed chronic active hepatitis A1/F1 before interferon therapy**.

A 46-year-old Japanese woman donated the right lobe of her liver to her 36-year-old brother, who had decompensated cirrhosis of unknown origin. She experienced mild liver dysfunction (117IU/L in alanine aminotransferase, normal range of 5 to 30IU/L) eight years before the donation. Her condition was diagnosed as chronic active HCV infection (serotype 2) on the basis of a liver biopsy and viral study that showed that her level of HCV-RNA was 13 kcopy/mL by real-time polymerase chain reaction analysis. The histological diagnosis showed chronic hepatitis A1/F1 (Figure 1). She received anti-viral therapy with intra-muscular interferon α-2b three times weekly for 24 weeks. Her serum HCV assay results were negative after two weeks of effective anti-viral therapy.

**Figure 2:**
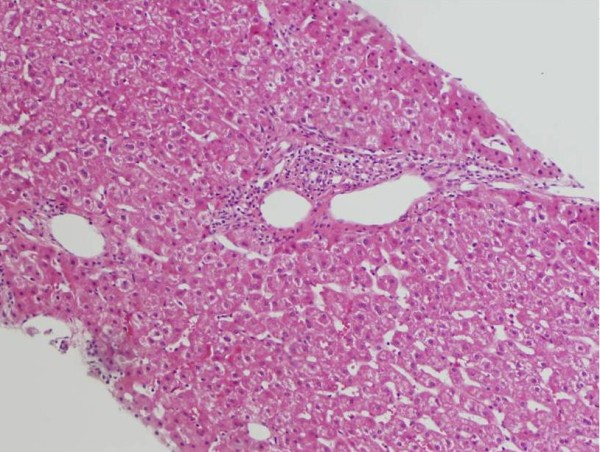
**A liver biopsy shows normal liver tissue without hepatitis before the liver donation**.

She was doing well and no HCV-RNA had been detected. She maintained an SVR without any complications until she was evaluated as a living donor. The donor evaluation revealed anti-HCV antibody, but her liver function test results were normal and HCV-RNA was negative by polymerase chain reaction analysis. She underwent an ultrasound-guided needle biopsy of her liver, and the pathological findings were normal and there were no findings of active hepatitis (Figure 2). She was approved as a living donor after a thorough evaluation by the ethics committee of the Nagasaki University Graduate School of Biomedical Sciences. She was discharged nine days after the liver donation. Her liver function recovered immediately. A computed tomography scan one year later showed that she had sufficient liver regeneration. Her brother was also doing well after the LT and had no HCV infection 40 months after surgery and no *de novo *malignancy.

## Discussion

Selecting a living donor in this case might be controversial, although a marginal donor can also sometimes be a candidate. The risks in this case included HCV transmission to the recipient, HCV reactivation in the recipient after LDLT, and donor risk during surgery. A number of studies have reported that the results with recipients of an HCV-infected graft were comparable to those of recipients of an HCV-negative graft [11-13]. The Scientific Registry of the United Network for Organ Sharing reported that the survival rate of 96 patients was significantly higher in the recipients of HCV-positive grafts than in recipients of HCV-negative grafts [13]. These results demonstrated that the use of an HCV-positive graft may also be acceptable in cadaveric LT. In contrast, an HCV-infected graft was not acceptable in LDLT. Patients who have HCV and who acquire an SVR after interferon therapy can be considered living donor candidates.

In this case, it was difficult to determine the indications for the donor selection before transplantation. Patients with serotype 2 HCV are more likely to achieve an SVR after interferon therapy than those with serotype 1. Our donor achieved an SVR of HCV. She and her brother were fully informed of the risk of peri-operative complications and the possibility that he would receive an HCV infection from the graft although she had obtained an SVR after anti-viral therapy. In theory, it is unlikely that a recipient would develop a viral infection from a graft that achieved an SVR.

## Conclusions

We report a case of LDLT from a donor previously treated with interferon for HCV. A patient with SVR status after interferon therapy might be considered a candidate for LDLT only if there are no other possibilities of LT for the recipient. A careful follow-up of the donor after donation is needed. The recipient also must have a very close follow-up because it is difficult to predict what might happen to the graft with post-transplant immunosuppression.

## Abbreviations

DDLT: deceased donor liver transplantation; HBV: hepatitis B virus; HCV: hepatitis C virus; LDLT: living donor liver transplantation; LT: liver transplantation; SVR: sustained viral response.

## Consent

Written informed consent was obtained from the patient for publication of this case report and any accompanying images. A copy of the written consent is available for review by the Editor-in-Chief of this journal.

## Competing interests

The authors declare that they have no competing interests.

## Authors' contributions

MH and SE shared responsibility for the management of this patient and were involved in drafting the manuscript or revising it critically for important intellectual content. MT shared responsibility for the management of this patient. All authors read and approved the final manuscript.
